# Applying the *SRL vs. ERL Theory* to the Knowledge of Achievement Emotions in Undergraduate University Students

**DOI:** 10.3389/fpsyg.2019.02070

**Published:** 2019-09-18

**Authors:** Jesús de la Fuente, José Manuel Martínez-Vicente, Francisco Javier Peralta-Sánchez, Angélica Garzón-Umerenkova, Manuel Mariano Vera, Paola Paoloni

**Affiliations:** ^1^School of Education and Psychology, University of Navarra, Pamplona, Spain; ^2^School of Psychology, University of Almería, Almería, Spain; ^3^Center of Research of Psychology, University of Almería, Almería, Spain; ^4^Fundacion Universitario Konrad Lorez, Bogotá, Colombia; ^5^Department of Personality, Assessment and Psychological Treatment, University of Granada, Granada, Spain; ^6^Río Cuato-CONICET National University, Córdoba, Argentina

**Keywords:** SRL vs. ERL theory, achievement emotions, burnout-engagement, university, stress

## Abstract

The *SRL vs*.ERL Theory predicts that a student's own self-regulation and the regulatory nature of the context are factors that jointly determine the student's level of motivational-affective variables. However, this principle has not yet been verified in the case of achievement emotions. The aim of this research was to test this prediction, with the hypothesis that students' level of self-regulation (low-medium-high), in interaction with the regulatory nature of the teaching (low-medium-high), would determine positive or negative emotions as well as the degree of burnout/engagement. A total of 440 university students completed validated questionnaires on self-regulation; regulatory teaching; achievement emotions in class, in study and in testing situations; and on burnout/engagement. Using a quasi-experimental design by selection, ANOVAs and MANOVAs (3 × 3; 5 × 1) were carried out. The results confirmed that the level of self-regulation and the level of external regulation jointly determined university students' level of achievement emotions, as well as their level of burnout/engagement. Based on these results, a five-level progressive scale was configured. We conclude that this scale may be useful and adequate as a heuristic technique or model for understanding and analyzing the type of student-teacher interaction that is taking place in the university classroom, and thereby learn the probability of stressful effects and the students' level of emotional health.

## Introduction

Classic Educational Psychology research on *individuals' learning variables* has focused on two large groups of constructs that would establish individual differences in learning and so predict achievement. On one hand is *intelligence*, with its related lines of research, such as the study of cognitive and metacognitive factors in learning processes. On the other hand is *personality*, as well as students' motivational-affective and meta-motivational processes. Detailed analysis over the past years has produced a considerable amount of research evidence, and a paradigm has emerged for the study of emotions and non-cognitive or “soft” skills in the educational sphere (Pekrun et al., 2009, 2019; Frenzel et al., 2016, 2018; Lüftenegger, 2016; Dicke et al., 2018; Muis et al., 2018). In a complementary fashion, research on *contextual variables of teaching* has analyzed the role of the teaching process and its elements, with special attention to the role of effective teaching (Pekrun et al., 2014a; Murayama et al., 2017; Gentsch et al., 2018; Mainhard et al., 2018). However, a precise analysis of the joint, interactive and interdependent relationships between the two sets of factors—pertaining to the learning process and the teaching process—remains to be achieved. Notwithstanding, certain interactive models have laid the foundation for this area of study (Vermunt, 1989, 2007; Bigg, 2001). Consequently, the present study aims to offer conceptual foundations and empirical evidence in this direction.

### Academic Emotions as a Learning Variable

#### Positive vs. Negative Academic Emotions

Emotions having to do with learning/achievement situations and outcomes are referred to as academic emotions (Pekrun et al., 2005, 2011, 2017a,b; Schutz and Pekrun, 2010; Pekrun and Stephens, 2012; Pekrun, 2014). Academic emotions therefore include achievement emotions experienced at school, but they also address emotions related to the instruction or the process of studying. Pekrun (1992) expanded on earlier conceptualizations of emotions by classifying academic emotions using a three-way taxonomy, namely, their *focus, valence, and activation*. Two types of academic or achievement emotions can be distinguished if we consider their origin: activity emotions originate in ongoing activities that relate to achievement, while outcome emotions stem from focusing on the outcomes of such activities (Pekrun, 2006). Both activity and outcome emotions are further classified by their valence (positive vs. negative or pleasant vs. unpleasant) and their role in activation (activating vs. deactivating). Students' activity emotions in academic settings have been addressed in recent research: for example, enjoyment as a positive, activating emotion (for an overview, see Ainley and Hidi, 2014) and boredom as a negative, deactivating emotion. Positive, activating emotions (enjoyment, hope, pride) are generally assumed to have positive effects on achievement, while negative (anger, anxiety, shame, hopelessness), and deactivating emotions (boredom, relief) would affect achievement and learning behavior in a negative fashion. This assumption is supported by empirical evidence (Frenzel et al., 2007; Pekrun et al., 2014b).

Empirical findings increasingly support that academic enjoyment and boredom are aligned with specific domains (Goetz et al., 2004, 2007a, 2014). Findings showed that enjoyment was the most domain-specific emotion, after comparing emotions assessed in six different subject domains. Adolescents' emotions in different subjects were shown to have relatively little relationship to each other; different levels of enjoyment and boredom were experienced in different subjects. While evidence increasingly confirms the domain specificity of academic emotions, little attempt has been made to search out the underlying mechanisms.

#### The Effect of Positive vs. Negative Emotions on Students

Several studies have reported positive effects of enjoyment on students' achievement (Pekrun, 2014; Pekrun et al., 2019), while boredom shows detrimental effects (Goetz et al., 2014), across scholastic domains. Motivation, meta-cognitive activities, and cognitive resources have been theorized as mediating factors. Students' mastery goals, interest, intrinsic motivation, attention, invested effort, self-regulation, elaboration and use of metacognitive strategies have been found in positive association with enjoyment, and in negative association with boredom; these elements have the same positive and negative associations with achievement (Goetz et al., 2007b).

In the academic context, we find enjoyment and boredom among the emotions most often reported (Goetz et al., 2007b; Linnenbrink-Garcia and Pekrun, 2011; Pekrun and Linnenbrink-Garcia, 2014). Because these two emotions are so prevalent in academic settings and so visible across academic domains (e.g., Goetz et al., 2007b; D'Mello, 2013), and because they affect learning and achievement in opposing directions, positive and negative emotions were selected as the object of the present study. From a complementary approach, the Transactional Analysis (TA) theory has also found a relationship between positive emotions and specific learning domains, in the teacher-student relations (Pishghadam and Khajavy, 2014).

Relationships of academic emotions with burnout vs. engagement have also been found. *Burnout* represents fatigue, depersonalization, lack of expectations and disaffection for a task (Maslach and Jackson, 1981); *engagement* represents taste, commitment and enjoyment with a given task (Maslach and Leiter, 1997). Previous research has reported factors that predict and probabilize both (Uludag and Yaratan, 2010). Thus, it has been found that academic emotions (positive vs. negative) are differentially associated with burnout (Burr and Dallaghan, 2019). It has also been found that engagement probabilizes metacognitive self-regulation and knowledge construction (Khosa and Volet, 2014). More recently, both have been conceptualized as positive (engagement) vs. negative (burnout) learning (Dormann et al., 2017). On the other hand, burnout has consistently appeared as a negative predictor of motivation and performance (Salanovaa et al., 2010; Stoeber et al., 2011), although the authors of the inventories, they have recognized that the direction between both constructs is not simple but complex, and requires more specific analysis through profiles (Leiter and Marlach, 2017a,b).

### Academic Emotions as Teaching Variable

#### Regulatory Teaching

Regulatory teaching is refers to encouragement of self-regulation in students and it's characteristic of effective teaching. In empirical research, high quality teachers are those who have a positive impact on their students' engagement with learning activities (Reeve et al., 2004). Some authors have explained self-regulation promoting teaching strategies (Paris and Winograd, 2003, pp. 12–14):

1. *Self-regulation can be taught with explicit instruction, directed reflection, and metacognitive discussions*. Cognitive research has shown that expertise can develop in many ways and explicit instruction is not always necessary. However, many children do not gain metacognitive insights or use SRL effectively without direct instruction and it seems plausible that many teachers can increase their own metacognitive understanding through explicit instruction. The most direct method of making new teachers aware of SRL is to incorporate it in the curriculum as a topic of study.

2. *Self-regulation can be promoted indirectly by modeling and by activities that entail reflective analyses of learning*. SRL can be taught indirectly with classroom activities, tools to evoke reflection and metacognitive understanding. One excellent method is the use of journals because they can be used with students of any age. Prospective teachers who use journals in classes learn to distinguish superficial entries and responses from analytic entries and responsive comments, so they are less likely to “do journals” as an activity and more likely to use journal writing as an avenue for self-exploration, self-discovery, and self-disclosure. A second tool that translates easily from teachers to students is conferences. Conferences can be focused on cooperative projects, report cards and grades, planning and brainstorming, and other classroom events but in all the endeavors, the focus of the conference can include analyses of thinking, learning, and teaching.

3*. Self-regulation can be promoted by assessing, charting, and discussing evidence of personal growth*. SRL can be promoted through record keeping of goals met, grades received, and progress made in behavior management and learning. Teachers who use these records will understand how periodic self-appraisal can lead to feelings of pride or to renewed efforts. This simple technique is often used by people who monitor their diets, exercise, expenditures, and so forth and it can easily be extended to academic performance.”

Recent research has shown that the perceived classroom learning environment variables were good predictors of students' self-regulation. Additionally, teacher variables (effectiveness teaching) were found to have direct relations with students' self-regulation and moderate the relationships between the learning environment and self-regulation variables (Yerdelen and Sungur, 2019).

#### The Influence of the Teaching Context on Students' Academic Emotions

Formerly, when researchers have attempted to predict students' academic emotions in social environments, they have relied mainly on parents' and teachers' observations, set by their own expectations, and their child raising or teaching practices, respectively. For example, Pekrun (2006) asserted that parents' and teachers' achievement expectations, and the structure of their interaction with the student (e.g., feedback practices, established goal structures, autonomy support vs. control) influences the source of the student's emotions (i.e., control- and value-related appraisals). The importance of social influences on students' enjoyment and boredom is specifically identified in other theoretical models (Goetz et al., 2007b). The social environment is considered a third type of predictor in model of academic boredom; how the subject domain is valued by teachers, parents and peers impacts the student's experiences of academic boredom. This perspective is adopted in the present study, where we apply it to the context of *regulatory teaching*.

### SRL vs. ERL Theory as Heuristic of Research

The theory of *Self-Regulated Learning vs. Externally-Regulated Learning* (de la Fuente, 2017) is founded on the following theoretical assumptions. Behavioral regulation of the individual can be defined as different types along a behavioral continuum:

#### Principle 1. Self-Regulated Behavior, Non-regulated Behavior or Dysregulated Behavior as a Personal Characteristic

(1) *Self-Regulation* (SR) has to do with positive proactivity, that is, the individual actively and adequately regulates and manages his or her own conduct. This level is referred to as *high level*, in terms of the degree to and quantity of behaviors used to regulate one's own behavior (level 3).

(2) *Non-Regulation* (NR) may be conceptually defined as a person's lack of proactivity, or the absence of self-regulating behaviors. This is the conceptual equivalent of *reactivity*. This level is referred to as *medium level*, in terms of the degree to and quantity of behaviors used to regulate one's own behavior (level 2).

(3) *Dysregulation* (DR) has to do with negative proactivity, that is, the individual actively but inadequately regulates and manages his or her own conduct. Examples include the use of self-handicapping strategies or procrastination. This level is referred to *low level*, in terms of the degree to and quantity of behaviors used to regulate one's own behavior (level 1). The three behavior types are shown in Table 1.

**Table 1:** Conceptual continuum and typologies of each self-regulatory behavior.

**Characteristics of the person**	**Self-regulation (SR)high level (3)**	**Non-regulation (NR) medium level (2)**	**Dys-regulation (DR) low level (1)**
	*Before* Self-analysis of tasks Self-defines goals Self-motivation	*Before* No analysis of tasks No goals No motivation	*Before* Erroneous self-analysis Erroneous goals Self-demotivation
	*During* Self-observation Self-analysis Self-correction	*During* No self-observation No supervision No self-correction	*During* Self-distraction Cognitive self-avoidance Self-handicapping Procrastination
	*After* Self-reflection Self-attributions Positive self-affect	*After* No reflection No attributions No affect	*After* Erroneous self-assessment Erroneous self-attributions Negative self-affect
*Type of Activity*	*Self-regulatory (SR)*	*Non-regulatory (NR)*	*Dys-regulatory (DR)*
Academic	Self-regulated learning (SRL)	No norms/limits	Self-handicapping

#### Principle 2. External Regulation, Non-regulation, or Dysregulation Provided by the Context

(1) *External Regulatory* (ER) context. Positive or adequate proactivity is promoted through the context, which clearly fosters self-regulation. This context features *high levels* (level 3) of external signs or encouragements to promote self-regulated behavior and increases its likelihood at each moment of learning acts: beginning, middle and end. Such encouragement can be in the form of *antecedents* (patterns, norms, limits, expectations of success in self-regulation, value given to self-regulation) or contextual *consequences* (positive and negative contingencies favoring self-regulation, adaptation, etc.).

(2) *External Non-Regulatory* (ENR) context. The context neither encourages self-regulation nor does it tend to dysregulate students' learning. *Medium level* or no external *signs* (level 2) or other aspects promote self-regulated behavior or dysregulated behavior, so as to make either of these more likely at the beginning, middle and end of learning acts. A *non-regulatory* context supposes that the individual would engage in a moderate level of self-regulated behavior, in the absence of contextual elements that enhance or discourage such action. The context is characterized by a lack of predictability of action.

(3) *External Dys-Regulatory* (EDR) context, actively promotes dysregulation or *low levels* of self-regulation (level 1). The context promotes proactivity that is not positive, but inadequate or negative. Many external signs make dysregulated behavior more likely, and encourage active dysregulation at the beginning, middle and end of learning acts. These signs can also be in the form of *antecedents* (modeling, rules, limits, expectations of success in self-regulation, value given to self-regulation) or contextual *consequences* (positive and negative contingencies, molding, etc.) that favor dysregulation. This kind of context would require the individual to make a great effort if self-regulation is pursued. The context is a strong predictor of negative action (see Table 2).

**Table 2:** Conceptual continuum of the context dimension, Externally-Regulated Learning (ERL).

**Characteristics of the Context**	**External regulation high level (3)**	**External non-regulation medium level (2)**	**External dys-regulation low level (1)**
	*Before* Presents analysis of tasks Suggests adjusted goals Suggests self-motivation	*Before* Does not present tasks Does not propose goals Does not induce motivation	*Before* Erroneous tasks Erroneous goals (Self-handicapping) Induces demotivation
	*During* Promotes self-observation Promotes self-analysis Self-correction	*During* No self-observation No supervision No self-correction	*During* Promotes self-distraction Cognitive self-avoidance, Self-handicapping, Procrastination
	*After* Promotes self-reflection Promotes adjusted self-attributions Promotes positive adjusted self-affect	*After* No reflection No attributions No affect	*After* Promotes erroneous self-assessment, Erroneous self-attributions. Promotes maladjusted self-affect
*Type of Context*	Externally-regulating	Non-regulating	Dys-regulating
Academic	Regulatory teaching (RT)	Laissez-faire	Stressful teaching

#### Principle 3. Academic Emotions as an Internally (SR) and Externally (ER) Mediated Process

According to this principle, academic emotions are produced in a probabilistic fashion, with both internal mediation (self-regulation as a personal characteristic) and external mediation (favoring or discouraging regulation). Human learning is thus envisioned as the *combination* of a person's self-regulating ability and the external regulatory features of the context, with four types of interactions. Self-regulated learning, therefore, may be explained and predicted by an individual's self-regulation in conjunction with the external characteristics of the context. Consequently, the prediction of the model is that the *1st combination* (low self-regulation and low externally-regulation) should produce few positive and many negative emotions, high burnout and low engagement. The *2nd combination* (low self-regulation and medium/high externally-regulation) should produce medium-low positive emotions and negative medium-high, medium-high burnout and medium-low engagement. The *3rd combination* (medium/high self-regulation and medium-low externally-regulation) should produce medium/high positive emotions and low negative emotions, medium-high engagement and medium-low burnout. The *4th combination* (high self-regulation and high externally-regulation) should produce high positive emotions and low negative emotions, high engagement and low burnout (see Table 3).

**Table 3:** Positive vs. negative emotions in the *SRL vs. ERL Theory*.

**Type of combination**	**Presage**	**Process (teaching)**	**Process (learning)**	**Product**	
Pintrich's journey metaphor	Driver	Highway	Driving	Positive vs. Negative Emotions	Success Arrival vs. Accident
Level	Self-Regulation (SR)^*^ (student)	Regulatory Teaching (ER)^*^(context)	Self-Regulated Learning (SRL) *(student)*	Achievement Emotions^*^(student)	Motivation^*^(student)
4°	High= > low stress	High= > low stress	High= > Deep approach Low = > Surface approach	High= > + emotions Low = > – emotions	High= > engagement Low = > burnout
3°	High= > low/medium stress	Low = > Medium/low stress	Medium/High= > Deep approach Medium/Low = > Surface approach	Medium/High= > + emotions Medium/Low = > – emotions	Medium/High= > engagement Medium/low = > burnout
2°	Low = > medium/high stress	High= > medium/high stress	Medium/Low = > Deep approach High/Medium> Surface approach	Moderate/Low = > + emotions Moderate/High= > – emotions	Moderate/Low = > engagement Medium/High= > burnout
1°	Low = > high stress	Low = > high stress	Low = > Deep approach High= > Surface approach	Low = > + emotions High= > – emotions	Low = > Engagement High= > Burnout

* *Variables of this research*.

### Aims and Hypothesis

Based on the foregoing models and empirical data, this investigation had several *objectives*: (1) to improve the heuristic technique for assessing the type of combination—as established by SRL vs. ERL Theory—using five types or levels; (2) to establish whether these interaction levels determined positive and negative achievement emotions, as defined in Pekrun's model; (3) to analyze whether there was a similar impact in the correlates of engagement and burnout. *Hypotheses* consistent with these objectives were defined as follows: (1) The possible combinations of student's level of self-regulation and level of external regulation offered by the teaching can be ordered in five progressive levels (averaging the level of personal self-regulation and the regulation promoted by the context, on a range between 1 and 3, and on a scale from 1 to 5); (2) the gradual increase of interaction level, ranging from 1 to 5, will lead to a proportionate increase in positive emotionality and a decrease in negative emotionality, as conceptualized by the Pekrun model; (3) using the same logic, these levels will correspond to a progressive increase in student engagement and a decrease in burnout.

## Method

### Participants

For the interdependence relations among low-medium-high levels of *Personal Self-Regulation* (SR), and *Regulatory Teaching* (RT), we used a total sample of 440 undergraduate students from two universities in Spain. A selected sample of 336 students was used to analyze the type of combination. The sample was composed of students enrolled in Psychology, Primary Education, and Educational Psychology degree programs; 86.5% were women and 13.5% were men. Their ages ranged from 19 to 49, with a mean age of 23.08 (σ_*X*_ = 4.4) years.

### Instruments (see Annex I. Complementary Material)

#### Learning Process

##### Personal self-regulation (meta-behavioral variable)

This variable was measured using the *Short Self-Regulation Questionnaire (SSRQ)* (Miller and Brown, 1991). It has already been validated in Spanish samples (Pichardo et al., 2014, 2018), and possesses acceptable validity and reliability values, similar to the English version. The Short SRQ is composed of four factors (goal setting-planning, perseverance, decision making and learning from mistakes) and 17 items (all of them with saturations >0.40), with a consistent confirmatory factor structure (Chi-Square = 250.83, *df* = 112, CFI = 0.95, GFI = 0.94, AGFI = 0.96, RMSEA = 0.05. *Internal consistency* was acceptable for the total of questionnaire items (α = 0.86) and for the factors of goal setting-planning (α = 0.79), decision making (α = 0.72) and learning from mistakes (α = 0.72). *Correlations* have been studied, between each item and its factor total, among the factors, and between each factor and the complete questionnaire, with good results in all cases, except for the decision-making factor, which had a lower correlation with other factors (range: 0.41–0.58). The correlations between the original version and the complete version, and between the original and the short versions with a Spanish sample (complete SRQ with 32 items and short SRQ with 17 items) are better for the short version (short-original: *r* = 0.85 and short-complete: *r* = 0.94; *p* < 0.01) than for the complete version (complete-original: *r* = 0.79; *p* < 0.01).

#### Teaching Process

##### Regulatory teaching (meta-instructional variable)

The Scales for Assessment of the Teaching-Learning Process, ATLP, student version (de la Fuente et al., 2012) were used to evaluate students' perception of the teaching process. The scale entitled *Regulatory Teaching* is Dimension 1 of the confirmatory model. IATLP-D1 comprises 29 items structured along five factors: Specific regulatory teaching, regulatory assessment, preparation for learning, satisfaction with the teaching, and general regulatory teaching. The scale was validated in university students (de la Fuente et al., 2012) and showed a factor structure with adequate fit indices (Chi-Square = 590.626; *df* = 48, *p* < 0.001, CF1 = 0.958, TLI = 0.959, NFI = 0.950, NNFI = 0.967; RMSEA = 0.068) and adequate internal consistency (IATLP D1: α = 0.83; Specific regulatory teaching, α = 0.897; regulatory assessment, α = 0.883; preparation for learning, α = 0.849; satisfaction with the teaching, α = 0.883 and general regulatory teaching, α = 0.883). The ATLP is a self-report instrument completed by the teacher and the students, available in Spanish and English versions. It also includes a qualitative part where students can make recommendations for improving each of the processes evaluated. As for the instrument's external validity, results are also consistent, since there are different interdependent relationships among perceptions of variables that exist in an academic environment.

#### Learning Product

##### Achievement emotions

The Achievement Emotions Questionnaire, AEQ (Pekrun et al., 2005) is a multidimensional self-report instrument designed to assess university students' achievement emotions. This questionnaire was generated on the basis of a quantitative and qualitative research program analyzing the emotions that students experienced in academic achievement situations. Several discrete emotions are measured within each of the three main academic achievement situations: attending class, studying, and completing tests and exams. The current version of the AEQ can measure eight class-related emotions, eight learning-related emotions, and eight test emotions. Three corresponding scales—class-related, learning-related, and test-related emotions—make up the three sections of the AEQ. Eighty items in the *class-related emotions scale* (CRE) measure the following eight emotions: class-related enjoyment, hope, pride, anger, anxiety, shame, hopelessness, and boredom. The *learning-related emotions scale* (LRE) contains 75 items and measures the same eight emotions in study situations. The *test emotions scale* (TES) contains 77 items that assess test-related enjoyment, hope, pride, relief, anger, anxiety, shame, and hopelessness. Each section is formed by three blocks of items, for assessment of emotions experienced either before, during, or after the achievement situations addressed in that section. Trait achievement emotions are assessed, that is, the student's typical, individual emotional reactions in achievement situations. The AEQ instructions can be altered for the purpose of measuring emotions experienced in a particular class subject (course-specific emotions), or in specific situations at a specific time (state achievement emotions).

The AEQ assesses four positive emotions (enjoyment, hope, pride, and relief) and five negative emotions (anger, anxiety, hopelessness, shame, and boredom). Two main criteria were used to decide which emotions to include. First, the emotions identified are frequently experienced by college students (Pekrun, 1992). Second, the emotions can be classified along two dimensions, each with two possible values: valence (positive vs. negative) and activation (activating vs. deactivating). Their combination results in four categories of emotions that can summarize how emotions affect learning, achievement, personality development, and health. Emotions are classified into the four categories as follows, *positive activating:* enjoyment, hope, pride; *positive deactivating*: relief; *negative activating:* anger, anxiety, shame; *negative deactivating:* hopelessness, boredom.

The three main types of achievement situations at university—attending class, studying, and taking tests and exams—differ according to function and social structure. This implies that emotions toward these situations would also differ. Enjoyment of classroom instruction, for example, would differ from enjoying the challenge of an exam. Some students may feel excited about going to class, others when taking exams. The AEQ takes this into account by providing separate scales for emotions that are class-related, learning-related, and test-related.

#### Confirmatory Factor Analysis and Reliability

(1) *Class-Related Emotions* (translation: Paoloni, 2014). The psychometric properties of the CRE were satisfactory in students from Spain. In this sample, the model obtained good fit indices. Unidimensionality of the scale and metric invariance were confirmed in the samples evaluated (Chi Square = 10,885,597, Degrees of freedom = 3052, *p* < 0.001; CFI = 0.951, TLI = 0.952, IFI = 0.963, TLI = 0.958, and CFI = 0.952; RMSEA = 0.041; HOELTER = 458, *p* < 0.05; 466 *p* < 0.01). The Cronbach alpha for this sample was 0.904, 0.803 (40 items), and 0.852 (40 items), for each part, respectively (80 items).

(2) *Learning-Related Emotions* (translation: de la Fuente, 2015a). The psychometric properties of the LRE were satisfactory in students from Spain. In this sample, the model obtained good fit indices. Unidimensionality of the scale and metric invariance were confirmed in the samples evaluated (Chi Square = 10885,597, Degrees of freedom = 3052, *p* < 0.001; CFI = 0.959, TLI = 0.942, IFI = 0.969, TLI = 0.955, and CFI = 0.958; RMSEA = 0.038; HOELTER = 501, *p* < 0.05; 511 *p* < 0.01). The Cronbach alpha for this sample was 0.930, 0.880 (38 items), and 0.846 (37 items), for each part, respectively (75 items).

(3) *Test-Related Emotions* (translation: de la Fuente, 2015b). The psychometric properties of the TRE were satisfactory in students from Spain. In this sample, the model obtained good fit indices. Unidimensionality of the scale and metric invariance were confirmed in the samples evaluated (Chi Square = 10885,597, Degrees of freedom = 3052, *p* < 0.001; CFI = 0.954, TLI = 0.946, IFI = 0.964, TLI = 0.959, and CFI = 0.953; RMSEA = 0.039; HOELTER = 492, *p* < 0.05; 502 *p* < 0.01). The Cronbach alpha for this sample was 0.913, 0.824, and 0.869, for each part, respectively (77 items).

##### Engagement-burnout

This version has shown adequate reliability and construct validity indices in a cross-cultural study.

*Engagement* was assessed with a validated Spanish version of the *Utrecht Work Engagement Scale for Students* (Shaufeli et al., 2002). The psychometric properties of the TRE were satisfactory in students from Spain. In this sample, the model obtained good fit indices. Unidimensionality of the scale and metric invariance were confirmed in the samples evaluated (Chi-square = 792,526, *df* = 74, *p* < 0.001; CFI = 0.954, TLI = 0.976, IFI = 0.954, TLI = 0.979, and CFI = 0.973; RMSEA = 0.083; HOELTER = 153, *p* < 0.05; 170 *p* < 0.01). The Cronbach alpha for this sample was 0.900 (14 items), 0.856 (7 items), and 0.786 (7 items), for each part, respectively.

*Burnout* was assessed with a validated Spanish version of the *Burnout Scale for Students* (Shaufeli et al., 2002). The psychometric properties of this version scale were satisfactory in students from Spain. In this sample, the model obtained good fit indices. Unidimensionality of the scale and metric invariance were confirmed in the samples evaluated (Chi Square = 767,885, *df* = 87, *p* < 0.001; CFI = 0.956, TLI = 0.964, IFI = 0.951, TLI = 0.951, and CFI = 0.953; RMSEA = 0.071; HOELTER = 224, *p* < 0.05; 246 *p* < 0.01). The Cronbach alpha for this sample was 0.874 (15 items), 0.853 (8 items), and 0.793 (7 items), for each part, respectively.

### Procedure

Participants voluntarily completed the scales using an online *platform* (de la Fuente et al., 2015a). A total of five specific teaching-learning processes in different university subjects, imparted over two academic years, were evaluated. *Presage* variables were evaluated in September to October of 2017 and 2018, *Process* variables in February to March of 2017 and 2018, and *Product* variables in May to June of 2017 and 2018. The procedure was approved by the respective Ethics Committees of the two universities, in the context of an R & D Project (2018–2020).

### Data Analysis

A previous confirmatory factor analysis was conducted in this sample as evidence of factorial validity and to ensure the previous structural fit of each inventory (Chi Square, NFI, TLI, RFI, RMSEA and HOELTER), using the statistical program AMOS (v. 22) Reliability was also calculated (Cronbach Alpha) through SPSS (v.25).

Using an ex-post-facto design, first, a 3 K-means cluster analysis was conducted to establish low-medium-high groups in each of the two variables: Personal Self-Regulation (SR) and Regulatory Teaching (RT). In the case of the SR variable, the values (Low = 2.70; Medium = 3.48; High = 4.20) formed the centers of the clusters, response ranges being low (1.00–3.09), medium (3.10–3.84), and high (3.85–5.00). In the case of the RT variable (Low = 2.72; Medium = 3.58; High = 4.34), formed the centers of the clusters, response ranges being low (1.00–2.34), medium (2.35–2.83) and high (2.84–5.00). In addition, several ANOVAs and MANOVAs were carried out, to ascertain the effect of low-medium-high levels of the dependent variable, achievement emotions. Also, using a 3-factor design (low-medium-high self-regulation levels) × 3 (low-medium-high levels of regulatory teaching), several MANOVAs were conducted, taking the aforementioned levels as the independent variable. Finally, based on the low-medium-high groups in both variables (SR and RT), five combinations were configured, according to the theoretical model proposed (see Table 4). MANOVAs were conducted to establish statistical suitability of these groupings, as well as the effects of the dependent variables defined, with Pillai's trace and Sheffé test index.

**Table 4:** Interdependence relations between the low-medium-high levels of *Self-Regulation* and *External Regulation (Regulatory Teaching)* as independent variables, in *achievement emotions*, burnout and engagement.

***DVs***	***Self-regulation***			***External regulation***		
	**1. *Low***	***2. Medium***	***3. High***	***1. Low***	***2. Medium***	***3. High***
	**DR (*n* = 104)**	**NR (*n* = 215)**	**SR (*n* = 99)**	**EDR (*n* = 85)**	**ENR (*n* = 173)**	**ER (*n* = 172)**
***Class Achievement Emotions (CAE)***						
*Positives (+)*	2.97 (0.59)	3.38 (0.54)	3.84 (0.61)^*^	3.11 (0.67)	3.29 (0.58)	3.72 (0.60)
*Negatives (–)*	2.43 (0.63)	2.05 (0.56)	1.69 (0.54)^*^	2.29 (0.64)	2.14 (0.62)	1.81 (0.55)^3 <2, 1^^*^
Enjoyment (+)	2.88 (0.63)	3.23 (0.62)	3.65 (0.72)^*^	2.99 (0.72)	3.13 (0.62)	3.58 (0.66)^*^
Hope (+)	2.98 (0.66)	3.51 (0.56)	4.03 (0.63)^*^	3.22 (0.73)	3.42 (0.64)	3.84 (0.64)^*^
Pride (+)	3.05 (0.70)	3.41 (0.62)	3.84 (0.68)^*^	3.11 (0.70)	3.29 (0.64)	3.80 (0.64)^*^
Boredom (–)	2.75 (0.87)	2.27 (0.79)	1.90 (0.80)^*^	2.60 (0.97)	2.39 (0.80)	1.91 (0.77)^*^
Anger (–)	2.19 (0.73)	1.86 (0.66)	1.57 (0.62)^*^	2.12 (0.74)	1.96 (0.67)	1.56 (0.54)^*^
Anxiety (–)	2.51 (0.72)	2.18 (0.65)	1.78 (0.62)^*^	2.24 (0.71)	2.21 (0.74)	1.97 (0.65) ^3 <2, 1^^*^
Shame (–)	2.57 (0.91)	2.19 (0.80)	1.79 (0.75)^*^	2.21 (0.83)	2.21 (0.88)	1.99 (0.74)
Hopelessness (–)	2.14 (0.74)	1.75 (0.60)	1.40 (0.55)^*^	1.85 (0.70)	1.86 (0.70)	1.50 (0.50) ^3 <2, 1^^*^
***Learning Achievement Emotions (LAE)***						
*Positives*	3.27 (0.61)	3.63 (0.53)	4.01 (0.57)^*^	3.36 (0.62)	3.55 (0.59)	3.98 (0.51)^*^
*Negatives*	2.61 (0.69)	2.14 (0.62)	1.80 (0.62)^*^	2.37 (0.80)	2.30 (0.69)	1.89 (0.56) ^3 <2, 1^^*^
Enjoyment (+)	3.13 (0.63)	3.46 (0.57)	3.90 (0.59)^*^	3.18 (0.82)	3.46 (0.68)	3.73 (0.57)^*^
Hope (+)	3.16 (0.74)	3.64 (0.66)	4.14 (0.68)^*^	3.34 (0.83)	3.56 (0.73)	4.01 (0.64) ^3>2, 1^^*^
Pride (+)	3.34 (0.76)	3.76 (0.63)	4.20 (0.63)^*^	3.11 (0.70)	3.29 (0.64)	3.80 (0.64)^*^
Boredom (–)	2.72 (0.88)	2.23 (0.83)	1.79 (0.75)^*^	2.60 (0.97)	2.39 (0.80)	1.91 (0.77) ^3 <2, 1^^*^
Anger (–)	2.29 (0.82)	1.96 (0.73)	1.59 (0.64)^*^	2.11 (0.81)	1.96 (0.67)	1.56 (0.54) ^1>2, 3^^*^
Anxiety (–)	2.97 (0.70)	2.57 (0.64)	2.27 (0.68)^*^	2.11 (0.71)	2.24 (0.74)	1.97 (0.65) ^3 <2, 1^^*^
Shame (–)	2.60 (0.82)	2.07 (0.76)	1.82 (0.76)^*^	2.21 (0.83)	2.21 (0.88)	1.99 (0.74) ^ns^
Hopelessness (–)	2.39 (0.90)	1.90 (0.72)	1.52 (0.71)^*^	1.85 (0.70)	1.86 (0.70)	1.50 (0.66) ^3 <2, 1^^*^
***Test Achievement Emotions (TAE)***						
*Positives (+)*	2.88 (0.68)	3.24 (0.60)	3.60 (0.62)^*^	3.00 (0.68)	3.15 (0.65)	3.51 (0.62)^*^
*Negatives (–)*	2.78 (0.56)	2.51 (0.55)	2.27 (0.56)^*^	2.61 (0.65)	2.58 (0.55)	2.44 (0.55) ^3 <2, 1^^*^
Enjoyment (+)	2.81 (0.70)	3.10 (0.68)	3.38 (0.81)^*^	2.87 (0.70)	3.08 (0.70)	3.32 (0.67)^*^
Hope (+)	2.87 (0.77)	3.32 (0.66)	3.74 (0.75)^*^	3.08 (0.73)	3.21 (0.78)	3.63 (0.68) ^3, 2>1^^*^
Pride (+)	2.96 (0.76)	3.33 (0.76)	1.70 (0.57)^*^	3.07 (0.70)	3.25 (0.72)	3.58 (0.75)^*^
Relief (–)	3.50 (0.85)	3.68 (0.75)	3.63 (0.88)	3.38 (0.84)	3.67 (0.76)	3.75 (0.82)^*^
Anger (–)	2.51 (0.70)	2.19 (0.68)	1.91 (0.67)^*^	2.24 (0.68)	2.25 (0.66)	1.99 (0.69) ^3 <2, 1^^*^
Anxiety (–)	3.28 (0.83)	2.90 (0.83)	2.60 (0.86)^*^	2.88 (0.94)	2.94 (0.90)	2.83 (0.85) ^n.s.^
Shame (–)	2.20 (0.87)	1.84 (0.74)	1.61 (0.75)^*^	2.01 (0.89)	1.96 (0.77)	1.72 (0.74) ^3 <2, 1^^*^
Hopelessness (–)	2.41 (0.88)	1.96 (0.79)	1.59 (0.78)^*^	2.07 (0.91)	2.04 (0.81)	1.72 (0.79) ^3 <2, 1^^*^
***Burnout*** **(–)**	2.61 (0.62)	2.20 (0.53)	1.88 (0.53)^*^	2.42 (0.68)	2.32 (0.58)	1.87 (0.56)^*^
Depletion	2.96 (0.82)	2.52 (0.54)	2.16 (0.59)^*^	2.69 (0.87)	2.62 (0.79)	2.34 (0.83)^*^
Cynicism	2.45 (0.93)	2.01 (0.81)	1.78 (0.75)^*^	2.25 (0.92)	2.17 (0.85)	1.78 (0.77)^*^
Lack of Effectiveness	2.43 (0.47)	2.08 (0.52)	1.71 (0.48)^*^	2.33 (0.65)	2.17 (0.52)	1.80 (0.51)^*^
***Engagement*** **(+)**	3.15 (0.63)	3.44 (0.60)	3.84 (0.60)^*^	3.20 (0.70)	3.37 (0.61)	3.38 (0.78)^*^
Vigor	2.86 (0.82)	3.23 (0.71)	3.69 (0.68)^*^	2.95 (0.73)	3.14 (0.73)	3.57 (0.73)^*^
Dedication	3.59 (0.77)	3.86 (0.69)	4.17 (0.18)^*^	3.62 (0.85)	3.77 (0.69)	4.18 (0.64)^*^
Absorption	3.00 (0.80)	3.22 (0.79)	3.67 (0.79)^*^	3.02 (0.88)	3.20 (0.80)	3.58 (0.77)^*^

*SR, Self-Regulation; NR, Non-Regulation; DR, Dys-Regulation; ER, External Regulation; ENR, External Non-Regulation; EDR, External Dys-Regulation*.

* *Statistical significance effect in each variable: p <0.001*.

## Results

### Interdependent Relations Among Levels of Personal Self-Regulation (SR) and Levels of Regulatory Teaching (RT) in the Achievement Emotions

#### Class Achievement Emotions (CAE)

A statistically significant main effect of the *SR IV (low-medium-high levels)* [*F*_(4, 714)_ = 14.831 (Pillai's Trace), *p* < 0.001, *n*^2^ = 0.077], and *RT IV (low-medium-levels)* [*F*_(4, 714)_ = 8.975 (Pillai's Trace), *p* < 0.001, *n*^2^ = 0.048], was noted on the CAE. The statistically significant partial effect was maintained of the *SR IV (low-medium-high levels)* for both *Positives Emotions* [*F*_(2, 365)_ = 25.945*, p* < 0.001, *n*^2^ = 0.127, 1>2>3], and *Negatives Emotions* [*F*_(2, 365)_ = 18.314 (Pillai's Trace), *p* < 0.001, n^2^ = 0.127; 3 >2 >1]. The statistically significant partial effect was maintained of the *PR IV (low-medium-high levels)* for both *Positive Emotions* [*F*_(2, 365)_ = 15.847*, p* < 0.001, *n*^2^ = 0.082, 3>2,1], and *Negative Emotions* [*F*_(2, 365)_ = 9.884 (Pillai's Trace), *p* < 0.001, *n*^2^ = 0.052; 3 <2,1]. No statistical effect of significant interaction appeared.

Complementarily, a statistically significant main effect of the *SR IV (low-medium-high levels)* [*F*_(16, 702)_ = 4.865 (Pillai's Trace), *p* < 0.001, *n*^2^ = 0.100], and *RT IV* (low-medium-levels) [*F*_(16, 702)_ = 3.804 (Pillai's Trace), *p* < 0.001, *n*^2^ = 0.080], was noted on the factors of CAE. The statistically significant partial effect was retained for *enjoyment* [*F*_(2, 366)_ = 5.385, *p* < 0.001, *n*^2^ = 0.037; *post-hoc*: 3>2>1], for *hope* [*F*_(2, 366)_ = 13.463, *p* < 0.001, *n*^2^ = 0.164; *post-hoc*: 3>2>1], for *pride* [*F*_(2, 366)_ = 15.540, *p* < 0.001, *n*^2^ = 0.080; *post-hoc*: 3>2>1], for *boredom* [*F*_(2, 366)_ = 9.749, *p* < 0.001, *n*^2^ = 0.952; *post-hoc*: 1>2>3], for *anger* [*F*_(2, 366)_ = 9.448, *p* < 0.001, *n*^2^ = 0.050; *post-hoc*: 1>2>3], for *anxiety* [*F*_(2, 366)_ = 13.033, *p* < 0.001, *n*^2^ = 0.068; *post-hoc*: 1>2>3], for *shame* [*F*_(2, 366)_ = 11.080, *p* < 0.001, *n*^2^ = 0.062; *post-hoc*: 1>2>3], and for *hopelessness* [*F*_(2, 366)_ = 17.667, *p* < 0.001, *n*^2^ = 0.090, *post-hoc*: 1 >2>3].

#### Learning Achievement Emotions (LAE)

A statistically significant main effect of the *SR IV (low-medium-high levels)* [*F*_(4, 696)_ = 16.145 (Pillai's Trace), *p* < 0.001, *n*^2^ = 0.085], and RT IV (low-medium-levels) [*F*_(4, 696)_ = 8.833 (Pillai's Trace), *p* < 0.001, *n*^2^ = 0.048], was noted on the LAE. The statistically significant partial effect was maintained of the *SR IV (low-medium-high levels)* for both *Positive Emotions* [*F*_(2, 348)_ = 27.716*, p* < 0.001, *n*^2^ = 0.135, 1 <2 <3], and *Negative Emotions* [*F*_(2, 348)_ = 21.804 (Pillai's Trace), *p* < 0.001, n^2^ = 0.111; 1>2>3]. The statistically significant partial effect was maintained of the *PR IV (low-medium-high levels)* for both *Positive Emotions* [*F*_(2, 348)_ = 15.028*, p* < 0.001, *n*^2^ = 0.079, 3>2,1], and *Negative Emotions* [*F*_(2, 348)_ = 8.205 (Pillai's Trace), *p* < 0.001, *n*^2^ = 0.045; 3 <2,1]. No statistical effect of significant interaction appeared.

Complementarily, a statistically significant main effect of the *SR IV (low-medium-high levels)* [*F*_(16, 684)_ = 4.943 (Pillai's Trace), *p* < 0.001, *n*^2^ = 0.104], and *RT IV* (low-medium-levels) [*F*_(16, 684)_ = 2.964 (Pillai's Trace), *p* < 0.001, *n*^2^ = 0.065], was noted on the factors of LAE. The statistically significant partial effect of *SR IV* was retained for *enjoyment* [*F*_(2, 348)_ = 18.713, *p* < 0.001, *n*^2^ = 0.097; *post-hoc*: 3>2>1], for *hope* [*F*_(2, 348)_ = 29.686, *p* < 0.001, *n*^2^ = 0.146; *post-hoc*: 3>2>1], for *pride* [*F*_(2, 348)_ = 17.887, *p* < 0.001, *n*^2^ = 0.093; *post-hoc*: 3>2>1], for *boredom* [*F*_(2, 348)_ = 15.194, *p* < 0.001, *n*^2^ = 0.080; *post-hoc*: 1>2>3], for *anger* [*F*_(2, 348)_ = 9.746, *p* < 0.001, *n*^2^ = 0.053; *post-hoc*: 1>2>3], for *anxiety* [*F*_(2, 348)_ = 16.603, *p* < 0.001, *n*^2^ = 0.097; *post-hoc*: 1>2>3], for *shame* [*F*_(2, 348)_ = 19.089, *p* < 0.001, *n*^2^ = 0.099; *post-hoc*: 1>2>3], and for *hopelessness* [*F*_(2, 348)_ = 19.308, *p* < 0.001, *n*^2^ = 0.100, *post-hoc*: 1 >2>3]. A statistically significant partial effect of *RT IV* was retained for *enjoyment* [*F*_(2, 348)_ = 9.841, *p* < 0.001, *n*^2^ = 0.054; *post-hoc*: 3>2,1], for *hope* [*F*_(2, 348)_ = 13,123, *p* < 0.001, *n*^2^ = 0.170; *post-hoc*: 3>2,1], for *pride* [*F*_(2, 348)_ = 13.693, *p* < 0.001, *n*^2^ = 0.073; *post-hoc*: 3>2>1], for *boredom* [*F*_(2, 348)_ = 13.165, *p* < 0.001, *n*^2^ = 0.070; *post-hoc*: 1,2>3], for *anger* [*F*_(2, 348)_ = 6.645, *p* < 0.001, *n*^2^ = 0.037; *post-hoc*: 1,2>3], for *anxiety* [*F*_(2, 348)_ = 3.090, *p* < 0.001, *n*^2^ = 0.037; *post-hoc*: 1>2>3], for *shame* [*F*_(2, 348)_ = 2.676, *p* < 0.001, *n*^2^ = 0.015; *post-hoc*: 1,2>3], and for *hopelessness* [*F*_(2, 348)_ = 7.935, *p* < 0.001, *n*^2^ = 0.044, *post-hoc*: 1,2>3].

#### Test Achievement Emotions (TAE)

A statistically significant main effect of the *SR IV (low-medium-high levels)* [*F*_(4, 716)_ = 14.276 (Pillai's Trace), *p* < 0.001, *n*^2^ = 0.074], and RT IV (low-medium-levels) [*F*_(4, 716)_ = 5.8705 (Pillai's Trace), *p* < 0.001, *n*^2^ = 0.032], was noted on the TAE. The statistically significant partial effect was maintained of the *SR IV (low-medium-high levels)* for both *Positive Emotions* [*F*_(2, 358)_ = 21.361*, p* < 0.001, *n*^2^ = 0.107, 3>2>1], and *Negative Emotions* [*F*_(2, 358)_ = 17.415 (Pillai's Trace), *p* < 0.001, n^2^ = 0.087; 1 >2 >3]. The statistically significant partial effect was maintained of the *PR IV (low-medium-high levels)* for both *Positive Emotions* [*F*_(2, 358)_ = 11.268*, p* < 0.001, *n*^2^ = 0.059, 3>2,1], and *Negative Emotions* [*F*_(2, 3585)_ = 0,595 (Pillai's Trace), ns, *n*^2^ = 0.052]. No statistical effect of significant interaction appeared.

Complementarily, a statistically significant main effect of the *SR IV (low-medium-high levels)* [*F*_(16, 704)_ = 4.613 (Pillai's Trace), *p* < 0.001, *n*^2^ = 0.095], and *RT IV* (low-medium-levels) [*F*_(16, 704)_ = 2.981 (Pillai's Trace), *p* < 0.001, *n*^2^ = 0.063], was noted on the factors of TAE. The statistically significant partial effect was retained for *enjoyment* [*F*_(2, 358)_ = 7.161, *p* < 0.001, *n*^2^ = 0.038; *post-hoc*: 3>2,1], for *hope* [*F*_(2, 358)_ = 11.813, *p* < 0.001, *n*^2^ = 0.062; *post-hoc*: 3>2,1], for *pride* [*F*_(2, 358)_ = 9.958, *p* < 0.001, *n*^2^ = 0.053; *post-hoc*: 3>2,1], for *relief* [*F*_(2, 358)_ = 4.789, *p* < 0.01, *n*^2^ = 0.952; *post-hoc*: 1>2,3], for *anger* [*F*_(2, 358)_ = 2.518, *p* < 0.05, *n*^2^ = 0.014; *post-hoc*: 1,2>3], for *anxiety* [*F*_(2, 358)_ = 0.341, ns, *n*^2^ = 0.002], for *shame* [*F*_(2, 358)_ = 0.225, ns, *n*^2^ = 0.001], and for *hopelessness* [*F*_(2, 358)_ = 2.405, *p* < 0.09 ns, *n*^2^ = 0.013].

#### Engagement-Burnout

A statistically significant general main effect of the *Self-Regulation IV* (*low-medium-high levels*) [*F*_(4, 1808)_ = 38.541 (Pillai's Trace), *p* < 0. 001, *n*^2^ = 0.079; *post-hoc*: 3 >2 > 1], and *Regulatory Teaching IV* (*low-medium-high levels*) [*F*_(4, 1808)_ = 21.850 (Pillai's Trace), *p* < 0. 001, *n*^2^ = 0.046; *post-hoc*: 3 >2 > 1] was observed on *Engagement-Burnout levels*. The statistically significant partial effect was maintained of *Self-Regulation* IV both *Engagement* [*F*_(2, 914)_ = 44.886, *p* < 0.001, *n*^2^ = 0.090, 1>2>3], and *Burnout* [*F*_(2, 914)_ = 76.096 (Pillai's Trace), *p* < 0.001, *n*^2^ = 0.144; 3 >2 >1]. A statistically significant general main effect of the *Regulatory Teaching IV (low-medium-high levels)* both *Engagement-Burnout* [*F*_(4, 1808)_ = 21.850, *p* < 0.001, *n*^2^ = 0.946, 1>2>3].

The combined analysis of the *Self-Regulation* IV'*s effect (low-medium-high levels)* on the components of *engagement-burnout* yielded a statistically significant main effect [*F*_(12, 1800)_ = 17535 (Pillai's Trace), *p* < 0.001, *n*^2^ = 0.105]. The statistically significant partial effect was retained for *vigor* [*F*_(2, 904)_ = 48.663, *p* < 0.001, *n*^2^ = 0.097; *post-hoc*: 3>2>1], for *dedication* [*F*_(2, 904)_ = 24.995, *p* < 0.001, *n*^2^ = 0.092; *post-hoc*: 3>2>1], for *absorption* [*F*_(2, 904)_ = 23.660, *p* < 0.001, *n*^2^ = 0.093; *post-hoc*: 3>2>1], for *exhaustion* [*F*_(2, 904)_ = 48.474, *p* < 0.001, *n*^2^ = 0.097; *post-hoc*: 1>2>3], for *cynicism* [*F*_(2, 904)_ = 30.573, *p* < 0.001, *n*^2^ = 0.063; *post-hoc*: 1>2>3], for *lack of effectiveness* [*F*_(2, 904)_ = 84.497, *p* < 0.001, *n*^2^ = 0.156; *post-hoc*: 1>2>3].

**Figure 1:**
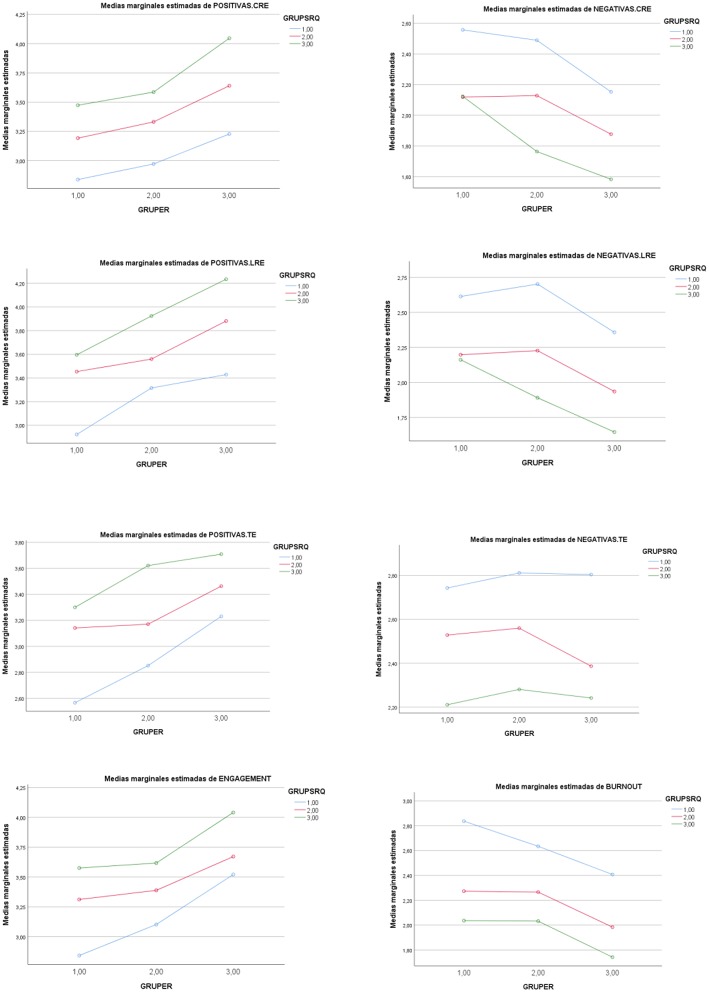
Graphic representation of the effect of the low (1)-medium (2)-high (3) levels in *Self- Regulation IV* (GRUPSRQ) and low (1)-medium (2)-high (3) in *Regulatory Teaching IV* (GRUPER) on the positive and negative academic emotions of each situation: class (CRE), study (LRE) and exam (TE). Complementarily, effects on ENGAGEMENT and BURNOUT.

In a complementary way, a statistically significant general main effect of the *Regulatory Teaching* IV (*low-medium-high levels*) was observed on the components of *engagement-burnout levels* [*F*_(12, 1800)_ = 9,218 (Pillai's Trace), *p* < 0. 001, *n*^2^ = 0.058]. The statistically significant partial effect was retained for *vigor* [*F*_(2, 904)_ = 35.222, *p* < 0.001, *n*^2^ = 0.072; *post-hoc*: 3>2>1], for *dedication* [*F*_(2, 904)_ = 33.156, *p* < 0.001, *n*^2^ = 0.068; *post-hoc*: 3,2>1], for *absorption* [*F*_(2, 904)_ = 21.111, *p* < 0.001, *n*^2^ = 0.041; *post-hoc*: 3,2>1], for *exhaustion* [*F*_(2, 904)_ = 21.111, *p* < 0.001, *n*^2^ = 0.145; *post-hoc*: 1, 2>3], for *cynicism* [*F*_(2, 904)_ = 17.524, *p* < 0.001, *n*^2^ = 0.037; *post-hoc*: 1,2>3], for *lack of effectiveness* [*F*_(2, 904)_ = 37.543, *p* < 0.001, *n*^2^ = 0.077 *post-hoc*: 1>2>3] (see Tables 4, 5, and Figure 1).

**Table 5:** Combined and Interdependent effects (3 × 3) between the independent variables of low-medium-high levels of *Self-Regulation (SR)* and low-medium-high levels of *Regulatory Teaching (RT)*, i.e., external regulation, on dependent variables (*n* = 201).

***SR***	***Low (n=87)***			***Medium (n=193)***			***High (n=86)***		
***RT*** ***n=***	***Low*** **24**	***Med*** **45**	***High*** **18**	***Low*** **43**	***Med*** **51**	***High*** **99**	***Low*** ***29***	***Med*** **47**	***High*** **86**
***Class Achievement Emotions (CAE)***									
Positive (+)	2.83 (0.60)	2.97 (0.58)	3.22 (0.64)	3.19 (0.59)	3.33 (0.46)	3.63 (0.53)	3.47 (0.51)	3.58 (0.67)	4.04 (0.51)^*^
Negative (–)	2.55 (0.62)	2.48 (0.58)	2.15 (0.74)	2.11 (0.54)	2.12 (0.59)	1.87 (0.18)	2.12 (0.84)	1.76 (0.44)	1.58 (0.50)^*^
Enjoyment (+)	2.75 (0.65)	2.87 (0.60)	3.19 (0.62)	3.06 (0.72)	3.19 (0.54)	3.48 (0.61)	3.34 (0.74)	3.32 (0.75)	3.87 (0.63)^*^
Hope (+)	2.83 (0.74)	3.01 (0.72)	3.22 (0.70)	3.33 (0.72)	3.46 (0.49)	3.73 (0.78)	3.71 (0.76)	3.82 (0.69)	4.21 (0.52)^*^
Pride (+)	2.91 (0.66)	3.02 (0.69)	3.26 (0.74)	3.17 (0.71)	3.33 (0.53)	3.69 (0.59)	3.36 (0.57)	3.61 (0.76)	4.04 (0.58)^*^
Boredom (–)	3.15 (0.19)	2.63 (0.69)	2.18 (0.73)	2.46 (0.89)	2.35 (0.69)	2.00 (0.80)	2.36 (0.88)	2.17 (0.93)	1.71 (0.69)^*^
Anger (–)	2.15 (0.91)	2.33 (0.72)	2.03 (0.83)	2.01 (0.78)	2.00 (0.74)	1.70 (0.53)	2.00 (0.98)	1.70 (0.69)	1.45 (0.45)^*^
Anxiety (–)	2.40 (0.62)	2.67 (0.68)	2.40 (0.97)	2.07 (0.65)	2.28 (0.69)	2.10 (0.56)	2.27 (0.99)	1.76 (0.47)	2.27 (0.99)^*^
Shame (–)	2.37 (0.75)	2.90 (0.90)	2.40 (0.99)	2.23 (0.85)	2.20 (0.79)	2.12 (0.69)	2.25 (0.99)	1.68 (0.64)	1.76 (0.69)^*^
Hopelessness (–)	2.27 (0.74)	2.16 (0.71)	1.88 (0.67)	1.78 (0.53)	1.84 (0.65)	1.55 (0.45)	1.69 (0.95)	1.45 (0.43)	1.33 (0.53)^*^
***Learning Achievement Emotions (LAE)***									
Positive (+)	2.92 (0.64)	3.31 (0.61)	3.42 (0.69)	3.45 (0.62)	3.55 (0.50)	3.88 (0.49)	3.59 (0.58)	3.92 (0.61)	4.23 (0.68)^*^
Negative (–)	2.81 (0.83)	2.70 (0.58)	2.35 (0.70)	2.39 (0.67)	2.22 (0.59)	1.93 (0.42)	2.16 (0.97)	1.89 (0.58)	1.64 (0.56)^*^
Enjoyment (+)	2.90 (0.56)	3.27 (0.56)	3.26 (0.66)	3.25 (0.65)	3.46 (0.54)	3.65 (0.49)	3.47 (0.72)	3.73 (0.74)	4.03 (0.44)^*^
Hope (+)	2.81 (0.75)	3.22 (0.70)	3.44 (0.75)	3.55 (0.78)	3.61 (0.60)	3.94 (0.54)	3.66 (0.59)	4.02 (0.68)	4.31 (0.62)^*^
Pride (+)	3.05 (0.79)	3.45 (0.72)	3.58 (0.83)	3.55 (0.64)	3.69 (0.60)	4.04 (0.59)	3.65 (0.63)	4.00 (0.62)	4.35 (0.57)^*^
Boredom (–)	2.91 (0.97)	2.72 (0.73)	2.28 (0.81)	2.48 (0.91)	2.30 (0.79)	1.88 (0.63)	2.28 (0.99)	1.90 (0.81)	1.57 (0.62)^*^
Anger (–)	2.35 (0.91)	2.33 (0.72)	2.03 (0.83)	2.01 (0.78)	2.00 (0.74)	1.70 (0.53)	2.00 (0.98)	1.70 (0.69)	1.45 (0.45)^*^
Anxiety (–)	2.85 (0.75)	3.80 (0.57)	2.83 (0.80)	2.52 (0.66)	2.62 (0.55)	2.49 (0.60)	2.47 (0.74)	2.36 (0.61)	2.13 (0.70)^*^
Shame (–)	2.49 (0.89)	2.80 (0.74)	2.47 (0.99)	2.03 (0.81)	2.16 (0.83)	1.96 (0.58)	2.07 (0.99)	1.82 (0.68)	1.67 (0.68)
Hopelessness (–)	2.44 (0.99)	2.56 (0.81)	2.16 (0.84)	1.92 (0.79)	2.03 (0.69)	1.62 (0.56)	1.98 (0.99)	1.64 (0.65)	1.39 (0.62)
***Test Achievement Emotions (TAE)***									
Positive (+)	2.56 (0.64)	2.85 (0.66)	3.23 (0.55)	3.14 (0.70)	3.27 (0.52)	3.46 (0.59)	3.30 (0.40)	3.62 (0.60)	3.70 (0.64)^*^
Negative (–)	2.74 (0.61)	2.81 (0.52)	2.80 (0.62)	2.52 (0.66)	2.55 (0.53)	2.38 (0.41)	2.21 (0.66)	2.28 (0.48)	2.24 (0.41)^*^
Enjoyment (+)	2.55 (0.69)	2.82 (0.71)	3.11 (0.56)	2.95 (0.80)	3.05 (0.59)	3.27 (0.67)	3.17 (0.40)	3.44 (0.66)	3.55 (0.62)^*^
Hope (+)	2.57 (0.65)	2.76 (0.76)	3.34 (0.68)	2.95 (0.80)	3.05 (0.89)	3.53 (0.64)	3.45 (0.47)	3.75 (0.70)	3.85 (0.80)^*^
Pride (+)	2.57 (0.65)	2.96 (0.72)	3.23 (0.63)	3.23 (0.72)	3.31 (0.59)	3.54 (0.73)	3.27 (0.56)	3.67 (0.60)	3.74 (0.71)^*^
Relief (–)	3.21 (0.80)	3.57 (0.77)	3.46 (0.91)	3.56 (0.71)	3.66 (0.66)	3.81 (0.85)	3.15 (0.65)	3.71 (0.77)	3.68 (0.76)^*^
Anger (–)	2.54 (0.64)	2.45 (0.60)	2.48 (0.81)	2.24 (0.68)	2.23 (0.67)	1.91 (0.53)	1.96 (0.77)	2.00 (0.56)	1.79 (0.58)
Anxiety (–)	3.15 (0.80)	3.35 (0.84)	3.43 (0.79)	2.90 (0.93)	2.91 (0.82)	2.85 (0.73)	2.51 (0.96)	2.61 (0.85)	2.57 (0.82)
Shame (–)	2.30 (0.86)	2.28 (0.85)	2.25 (0.99)	1.29 (0.89)	1.90 (0.67)	1.67 (0.62)	1.58 (0.87)	1.52 (0.53)	1.62 (0.68)
Hopelessness (–)	2.55 (0.76)	2.38 (0.84)	2.37 (0.93)	2.02 (0.91)	2.07 (0.72)	1.68 (0.65)	1.84(1.00)	1.54 (0.54)	1.52 (0.81)
***Engagement*** **(+)**	2.87 (0.70)	3.10 (0.60)	3.50 (0.57)	3.31 (0.67)	3.39 (0.57)	3.68 (0.57)	3.58 (0.64)	3.62 (0.60)	4.00 (0.50)^*^
Vigor	2.59 (0.70)	2.79 (0.68)	3.26 (0.73)	3.06 (0.69)	3.18 (0.68)	3.46 (0.73)	3.40 (0.66)	3.49 (0.68)	3.86 (0.80)^*^
Dedication	3.19 (0.69)	3.59 (0.75)	3.93 (0.73)	3.81 (0.81)	3.79 (0.64)	4.13 (0.59)	3.85 (0.90)	3.95 (0.65)	4.13 (0.70)^*^
Absorption	2.83 (0.86)	2.93 (0.77)	3.31 (0.76)	3.06 (0.84)	3.20 (0.77)	3.44 (0.79)	3.39 (0.89)	3.47 (0.76)	3.86 (0.70)^*^
***Burnout*** **(–)**	2.84 (0.53)	2.64 (0.61)	2.40 (0.65)	2.29 (0.68)	2.27 (0.48)	1.98 (0.47)	2.05 (0.57)	2.03 (0.55)	1.74 (0.44)^*^
Depletion	3.17 (0.74)	2.95 (0.79)	2.85 (0.87)	2.59 (0.90)	2.55 (0.72)	2.36 (0.77)	2.22 (0.66)	2.26 (0.79)	2.07 (0.74)^*^
Cynism	2.61 (0.82)	2.52 (0.95)	2.20 (0.99)	2.13 (0.95)	2.07 (0.73)	1.73 (0.65)	2.75 (0.60)	2.45 (0.50)	2.16 (0.53)^*^
Lack of effect	2.75 (0.60)	2.45 (0.50)	2.16 (0.53)	2.19 (0.58)	2.14 (0.49)	1.86 (0.45)	1.96 (0.65)	1.87 (0.44)	1.55 (0.40)^*^

* *Statistical effect in the variables*.

*SR, Self-Regulation Levels; RT, Regulatory Teaching Levels*.

### Combined Effects of Regulation Variables: A Utility Model™ for Types of Interactions Between Levels of Self-Regulation and External Regulation (Regulatory Teaching)

#### Building a Combination Typology for Understanding Academic Emotions and Effects

The multivariate analyses (MANOVAs) showed a statistically significant main effect of the five interaction types on the low-medium-high levels of SR and of RT (see Table 6):

**Table 6:** Combination between the parameters of the model hypothesized by the SRL vs. ERL Theory: the *Utility Model*™ (de la Fuente, 2019).

**Combination level**		**Regulation average/rank**		**Regulation tendency rank**	**Academic Emotions**			**ENG vs. BURN**
**SR level (range)**	**RT level (range)**			**>**		**<**	
**3** (3.85–5.00) **H**	**3** (2.84–5.00) **H**	3.0	**5**	High-High: *High-Regulation*	*++*		–	*High ENG*
**2** (3.10–3.84) **M**	**3** (2.84–5.00) **H**	2.5	**4**	Medium-High: *Regulation*	+		–	*M-H ENG*
**3** (3.85–5.00) **H**	**2** (2.35–2.83) **M**	2.5	**4**	High-Medium: *Regulation*	+		–	*M-H ENG*
**2** (3.10–3.84) **M**	**2** (2.35–2.83) **M**	2.0	**3**	Medium: *Non-Regulation*	+	*=*	–	*M EN/BU*
**2** (3.10–3.84) **M**	**1** (1.00–2.34) **L**	1.5	**2**	Medium-Low: *Dys-regulation*	–		+	*M-H BUR*
**1** (1.00–3.09) **L**	**2** (2.35–2.83) **M**	1.5	**2**	Low-Medium: *Dys-regulation*	–		+	*M-H BUR*
**1** (1.00–3.09) **L**	**1** (1.00–2.34) **L**	1.0	**1**	Low-Low: *High Dys-regulation*	–	–	+	*High BUR*

*H, high; M, medium; L, low*.

*Combination 1* presented a statistically significant low level in *SR* and low level in *RT (1 and 1)*. The **average regulation level of 1.0**, and the **rank level** is **1**. The range of regulation tends toward low SR and low RT, associated with a *high level of dysregulation*. The most probable emotions are low levels of positive emotions and high levels of negatives emotions. Consequently, the effects are a *high level of stress: high burnout and low engagement*.

*Combination 2* had a statistically significant low-medium level in *SR* and medium-low level in *RT* and vice versa *(2 and 1, or 1 and 2)*. The **average regulation level is 1.5**, and the **rank level** is **2**. The range of regulation tends toward low-medium SR and low-medium RT, and vice versa, associated with *medium-low level of dysregulation*. The most probable emotions are medium-low level of positive emotions and medium-low level of negative emotions. Consequently, the effects are a *medium-high level of stress: medium-high burnout and medium-low engagement*.

*Combination 3* presented a statistically significant medium SR level *(2)* and medium RT level *(2 and 2)*. The **average regulation level of 2.0**, and the **rank level** is **3**. The range of regulation tends toward medium SR and medium RT, associated with *medium level of dysregulation*. The most probable emotions are medium level of positive emotions and medium level of negative emotions. Consequently, the effects are a *medium level of stress: medium burnout and medium engagement*.

*Combination 4* had a statistically significant medium SR- high RT and high RT- medium SR (*2 and 3, or 3 and 2*). The **average regulation level is 2.5**, and the **rank level** is **4**. The range of regulation tends toward high SR–medium RT and medium SR and high RT, associated with a good level of *regulation*. The most probable emotions are medium-high level of positive emotions and medium-low level of negative emotions. Consequently, the effects are a *medium-low level of stress: medium-low burnout and medium-high engagement*.

*Combination 5* presented statistically significant high SR- high RT and high RT- high *SR (3 and 3)*. The **average regulation level is 3.0**, and the **rank level** is **5**. The range of regulation tends toward high SR–high RT, associated with a *high level* of *regulation*. The most probable emotions are high level of positive emotions and low level of negative emotions. Consequently, the effects are a *low level of stress: low engagement and high burnout*.

#### Empirical Evidence for Combination Typology in Understanding Achievement Emotions

##### Preliminary analysis

The MANOVA produced statistically significant differences among the five groups in levels of self-regulation (SR) and regulatory teaching (RT); both variables were adequately configured as established in Table 6. See Table 7 for statistical effects.

**Table 7:** Combined Effects of Levels in Regulatory Type variables (5 × 2; 5 × 8): Mean score, standard deviation and specific effects (*n* = 336).

**DVs**	**Type of Combination in Groups (IVs)**						
	**1 (*n* = 24)**	**2 (*n* = 88)**	**3 (*n* = 119)**	**4 (*n* = 88)**	**5 (*n* = 47)**	**Effects** ***Post hoc***	
*Configuration Group*						*F*_(8, 2500)_ = 187.65 (Pillay), *p* < 0.001, *n*^2^ = 0.423	
*Self-Regulation*	2.65 (0.37)	3.02 (0.42)	3.41 (0.44)	3.80 (0.39)	4.23 (0.29)	*F*_(4, 1025)_ = 302.61, *p* < 0.001, *n*^2^ = 0.541, all *p* < 0.001	
*Regulatory Teaching*	2.73 (0.32)	3.24 (0.50)	3.63 (0.68)	4.03 (0.44)	4.39 (0.29)	*F*_(4, 1025)_ = 252.64, *p* < 0.001, *n*^2^ = 0.496, all *p* < 0.001	
***Class Achievement Emotions (CAE)***						*F*_(12, 1083)_ = 11.127, *p* < 0.001, *n*^2^ = 0.110	
*Positive* (+)	2.83 (0.60)	3.07 (0.59)	3.32 (0.50)	3.62 (0.58)	4.04 (0.51)*	*F*_(4, 361)_ = 33.378, *p* < 0.001, *n*^2^ = 0.270; 5,4>3>2,1, *p* < 0.001	
*Negative (–)*	2.40 (0.59)	2.24 (0.61)	2.08 (0.66)	1.78 (0.44)	1.55 (0.51)*	*F*_(4, 361)_ = 17.461, *p* < 0.001, *n*^2^ = 0.162; 5,4 <3 <2,1, *p* < 0.001	
						*F*_(32, 1428)_ = 5,483, *p* < 0.001, *n*^2^ = 0.109	
Enjoyment (+)	2.75 (0.65)	2.96 (0.67)	3.20 (0.57)	3.44 (0.66)	3.87 (0.63)*	*F*_(4, 361)_ = 21,165, *p* < 0.001, *n*^2^ = 0.190, 5,4>3>2,1, *p* < 0.001	
Hope (+)	2.83 (0.74)	3.16 (0.63)	3.45 (0.68)	3.76 (0.62)	4.21 (0.52)*	*F*_(4, 361)_ = 34,882, *p* < 0.001, *n*^2^ = 0.278 5,4,3, 2>1, *p* < 0.001	
Pride (+)	2.91 (0.66)	3.10 (0.70)	3.32 (0.56)	3.67 (0.65)	4.04 (0.58)*	*F*_(4, 361)_ = 25,344, *p* < 0.001, *n*^2^ = 0.219, 5>4,3,2>1, *p* < 0.001	
Boredom (–)	3.15 (0.96)	2.54 (0.79)	2.32 (0.16)	2.05 (0.84)	1.71 (0.69)*	*F*_(4, 361)_ = 18,064, *p* < 0.001, *n*^2^ = 0.167, 1,2>3>4,5 *p* < 0.001	
Anger (–)	2.57 (0.79)	2.05 (0.65)	1.94 (0.69)	1.64 (0.53)	1.40 (0.51)*	*F*_(4, 361)_ = 18.757, *p* < 0.001, *n*^2^ = 0.162, 1,2>3>4,5, *p* < 0.001	
Anxiety (–)	2.40 (0.62)	2.38 (0.71)	2.30 (0.76)	1.99 (0.56)	1.70 (0.62)*	*F*_(4, 361)_ = 10.904, *p* < 0.001, *n*^2^ = 0.108, 1,2>3>4,5, *p* < 0.001	
Shame (–)	2.37 (0.75)	2.57 (0.93)	2.24 (0.85)	1.97 (0.70)	1.76 (0.69)*	*F*_(4, 361)_ = 10.063, *p* < 0.001, *n*^2^ = 0.100, 1,2>3>4,5, *p* < 0.001	
Hopelessness (–)	2.27 (0.74)	1.98 (0.65)	1.83 (0.69)	1.52 (0.44)	1.33 (0.57)*	*F*_(4, 361)_ = 16.097, *p* < 0.001, *n*^2^ = 0.151, 1,2>3>4,5, *p* < 0.001	
***Learning Achievement Emotions (LAE)***						*F*_(8, 704)_ = 16.283, *p* < 0.001, *n*^2^ = 0.156	
*Positive* (+)	2.92 (0.77)	3.37 (0.62)	3.53 (0.54)	3.89 (0.54)	4.23 (0.48)*	*F*_(4, 352)_ = 10.327, *p* < 0.001, *n*^2^ = 0.266; 5>4>3,2>1, *p* < 0.001	
*Negative (*–*)*	2.61 (0.83)	2.49 (0.66)	2.24 (0.65)	1.91 (0.51)	1.64 (0.54)*	*F*_(4, 361)_ = 8.209, *p* < 0.001, *n*^2^ = 0.190; 5,4>3>2,1, *p* < 0.001	
						*F*_(32, 1392)_ = 4,292, *p* < 0.001, *n*^2^ = 0.090	
Enjoyment (+)	2.93 (0.55)	3.26 (0.59)	3.40 (0.59)	3.68 (0.59)	4.02 (0.47)*	*F*_(4, 352)_ = 22.131, *p* < 0.001, *n*^2^ = 0.202, 5,4>3,2>1, *p* < 0.001	
Hope (+)	2.78 (0.73)	3.36 (0.74)	3.52 (0.62)	3.96 (0.60)	4.27 (0.66)*	*F*_(4, 352)_ = 30,794, *p* < 0.001, *n*^2^ = 0.259, 5,4>3,2>1, *p* < 0.001	
Pride (+)	3.02 (0.77)	3.48 (0.69)	3.66 (0.65)	4.00 (0.61)	4.30 (0.62)*	*F*_(4, 352)_ = 24,021, *p* < 0.001, *n*^2^ = 0.214, 5,4 >3,2>1, *p* < 0.001	
Boredom (–)	2.93 (0.94)	2.62 (0.81)	2.26 (0.80)	1.88 (0.69)	1.58 (0.64)*	*F*_(4, 352)_ = 22.311, *p* < 0.001, *n*^2^ = 0.202, 1,2>3>4,5, *p* < 0.001	
Anger (–)	2.39 (0.89)	2.19 (0.76)	1.99 (0.77)	1.73 (0.58)	1.45 (0.76)*	*F*_(4, 352)_ = 12,604, *p* < 0.001, *n*^2^ = 0.125, 1,2>3>4,5, *p* < 0.001	
Anxiety (–)	2.88 (0.62)	2.85 (0.66)	2.63 (0.62)	2.45 (0.61)	2.12 (0.69)*	*F*_(4, 352)_ = 11,656, *p* < 0.001, *n*^2^ = 0.117, 1,2>3,4 >5, *p* < 0.001	
Shame (–)	2.49 (0.83)	2.48 (0.85)	2.21 (0.83)	1.61 (0.93)	1.67 (0.68)*	*F*_(4, 352)_ = 11,714, *p* < 0.001, *n*^2^ = 0.117, 1,2>3>4,5, *p* < 0.001	
Helplessness (–)	2.44 (0.99)	2.29 (0.86)	2.05 (0.75)	1.63 (0.60)	1.38 (0.62)*	*F*_(4, 352)_ = 17,632, *p* < 0.001, *n*^2^ = 0.167, 1,2>3>4,5, *p* < 0.001	
***Learning Achievement Emotions (TAE)***						*F*_(8, 724)_ = 11,175, *p* < 0.001, *n*^2^ = 0.110	
*Positive* (+)	2.56 (0.64)	2.97 (0.69)	3.19 (0.51)	3.52 (0.58)	3.70 (0.64)*	*F*_(4, 362)_ = 22,124, *p* < 0.001, *n*^2^ = 0.196; 5,4>3>2,1, *p* < 0.001	
*Negative (–)*	2.74 (0.61)	2.68 (0.60)	2.57 (0.58)	2.34 (0.38)	2.24 (0.51)*	*F*_(4, 362)_ = 8,259, *p* < 0.001, *n*^2^ = 0.084; 5,4 <3 <2,1, *p* < 0.001	
						*F*_(32, 1432)_ = 3,590, *p* < 0.001, *n*^2^ = 0.074	
Enjoyment (+)	2.53 (0.71)	2.88 (0.72)	3.09 (0.56)	3.34 (0.68)	3.52 (0.63)*	*F*_(4, 362)_ = 13.866, *p* < 0.001, *n*^2^ = 0.133, 5,4>3>2,1, *p* < 0.001	
Hope (+)	2.58 (0.69)	2.96 (0.78)	3.26 (0.59)	3.63 (0.67)	3.85 (0.76)*	*F*_(4, 362)_ = 23.574, *p* < 0.001, *n*^2^ = 0.207, 5,4>3>2>, *p* < 0.001	
Pride (+)	2.58 (0.68)	3.08 (0.73)	3.23 (0.58)	3.59 (0.52)	3.75 (0.71)*	*F*_(4, 362)_ = 18.643, *p* < 0.001, *n*^2^ = 0.171, 5,4>3>2,1, *p* < 0.001	
Relief (–)	3.15 (0.82)	3.57 (0.74)	3.57 (0.80)	3.77 (0.82)	3.68 (0.78)*	*F*_(4, 362)_ = 2.736, *p* < 0.05, *n*^2^ = 0.029, 1>4,5 *p* < 0.05	
Anger (–)	2.54 (0.64)	2.36 (0.64)	2.25 (0.71)	1.95 (0.58)	1.79 (0.58)*	*F*_(4, 362)_ = 10.643, *p* < 0.001, *n*^2^ = 0.105, 1,2>3>4,5, *p* < 0.001	
Anxiety (–)	3.15 (0.80)	3.16 (0.90)	2.98 (0.86)	2.76 (0.78)	2.57 (0.82)*	*F*_(4, 362)_ = 5.130, *p* < 0.001, *n*^2^ = 0.054, 1,2>3>4,5, *p* < 0.001	
Shame (–)	2.30 (0.86)	2.11 (0.89)	1.94 (0.80)	1.60 (0.59)	1.52 (0.68)*	*F*_(4, 362)_ = 7.778, *p* < 0.001, *n*^2^ = 0.079, 1,2>3>4,5, *p* < 0.001	
Helplessness (–)	2.55 (0.76)	2.22 (0.88)	2.11 (0.80)	1.62 (0.61)	1.52 (0.51)*	*F*_(4, 361)_ = 13.824, *p* < 0.001, *n*^2^ = 0.133, 1,2>3>4,5, *p* < 0.001	
						*F*_(8, 1816)_ = 30.135 *p* < 0.001, *n*^2^ = 0.130	
***Engagement*** **(+)**	2.84 (0.62)	3.18 (0.63)	3.42 (0.47)	3.64 (0.59)	4.04 (0.51)*	*F*_(4, 908)_ = 61.006 *p* < 0.001, *n*^2^ = 0.212, 5>4>3>2>1 *p* < 0.001	
***Burnout*** **(–)**	2.83 (0.55)	2.49 (0.67)	2.26 (0.43)	2.00 (0.50)	1.74 (0.47)*	*F*_(4, 908)_ = 60.421 *p* < 0.001, *n*^2^ = 0.210, 5 <4 <3 <2 <1 *p* < 0.001	
						*F*_(24, 3624)_ = 13.425, *p* < 0.001, *n*^2^ = 0.082	
Vigor (+)	2.55 (0.70)	2.89 (0.70)	3.21 (0.68)	3.46 (0.71)	3.87 (0.61)*	*F*_(4, 908)_ = 58.317, *p* < 0.001, *n*^2^ = 0.204, 1,2 <3 <4,5, *p* < 0.001	
Dedication (+)	3.18 (0.70)	3.68 (0.76)	3.82 (0.68)	4.02 (0.61)	4.26 (0.61)*	*F*_(4, 908)_ = 36.020, *p* < 0.001, *n*^2^ = 0.137, 1,2 <3 <4,5, *p* < 0.001	
Absorption(+)	2.79 (0.87)	2.96 (0.80)	3.24 (0.78)	3.45 (0.79)	3.87 (0.70)*	*F*_(4, 908)_ = 33.448, *p* < 0.001, *n*^2^ = 0.129, 1,2 <3 <4,5, *p* < 0.001	
Depletion (–)	3.13 (0.72)	2.78 (0.87)	2.60 (0.76)	2.30 (0.76)	2.07 (0.75)*	*F*_(4, 908)_ = 20.831, *p* < 0.001, *n*^2^ = 0.113, 1,2>3>4,5, *p* < 0.001	
Cynism (–)	2.62 (0.84)	2.35 (0.97)	2.09 (0.80)	1.83 (0.71)	1.59 (0.80)*	*F*_(4, 908)_ = 27.498, *p* < 0.001, *n*^2^ = 0.108, 1,2>3>4,5, *p* < 0.001	
Lack of effect (–)	2.75 (0.59)	2.33 (0.55)	2.11 (0.50)	1.87 (0.45)	1.56 (0.40)*	*F*_(4, 908)_ = 80.415, *p* < 0.001, *n*^2^ = 0.262, 1,2>3>4,5, *p* < 0.001	

*Type 1 (Low Self-Regulation, and Low Regulatory Teaching); Type 2 (Low Self-Regulation and High Regulatory Teaching); Type 3 (Medium Self-Regulation and Medium Regulatory Teaching); Type 4 (High Self-Regulation and Low Regulatory Teaching); Type 5 (High Self-Regulation and High Regulatory Teaching). For more information, see Table 6*.

##### Effects

**Figure 2:**
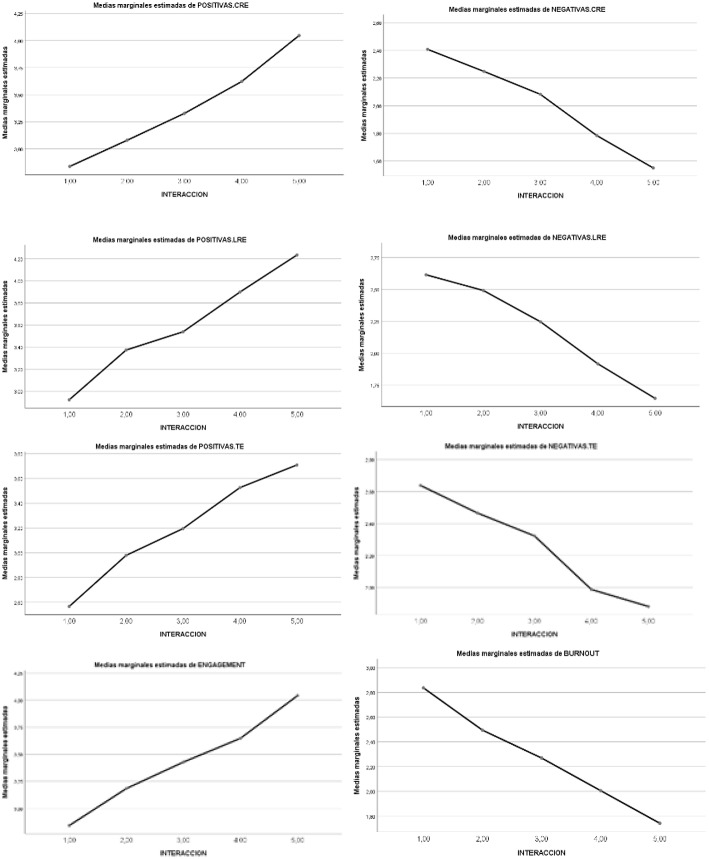
Graphic representation of the effects of the five types of interactions (levels 1–5) in *achievement emotions* (positive and negative) and in engagement-burnout, in the three situations: class (CRE), study (LRE), and exam (TAE).

A statistically significant main effect of the *five combinations of SR and RT as IV* was noted in *Class Achievement Emotions (CAE), Learning Achievement Emotions (LAE)* and *Test Achievement Emotions (TAE)*. The statistically significant partial effect was maintained of the *five combinations of SR and RT IV* for both *Positive Emotions* and *Negative Emotions*. In the case of *positive emotions*, a significant statistical effect appeared in favor of higher levels [4, 5 > 3 > 2, 1], while for *negative emotions* the effect was reversed, in favor of lower levels [1, 2 > 3 > 4, 5]. The statistically significant partial effect was maintained for each positive emotion (*enjoyment, hope, pride)*, and for negative emotions (*boredom -or relief-, anger, anxiety, shame*, and *hopelessness)*. Complementarily, in the case of *engagement*, a significant statistical effect appeared in favor of higher levels [4, 5 > 3 > 2, 1], while for *burnout* the effect was reversed, in favor of lower levels [1, 2 > 3 > 4, 5]. The statistically significant partial effect was maintained for *engagement* factors (*vigor, dedication, absorption)*, and for *burnout* factors (*depletion, cynicism, and lack of effectiveness)* (see Table 7). The graphic representation of the differential progressive effect of the combination between SR and RT levels is shown in Figure 2. Thus, while positive academic emotions and engagement progressively increase through the 5 levels of interaction, negative academic emotions and burnout decrease in the same proportion. Specifically, the clearest effects are increased *vigor* as the degree of interaction rises, and greater *loss of effectiveness* with lower interaction levels.

## Discussion

SRL vs. ERL Theory (de la Fuente, 2017) predicts that achievement emotions may be determined jointly by the students' degree of *self-regulation* and the level of *external regulation* offered by the teaching process. Furthermore, this type of interaction can be understood as the combination of *low-medium-high levels* of both factors, as seen in prior evidence (de la Fuente et al., 2015b, 2017). This hypothesis, however, has not been tested in reference to achievement emotions, even though there is recent research that considers this focus (Frenzel et al., 2018).

In the case of the *first hypothesis*, the evidence presented here shows the plausibility of ordering the combinations of students' levels of self-regulation (low-medium-high) and the regulatory level of the teaching process (low-medium-high), along a continuum. This allows for an improved combination model that organizes this interactive reality, as compared to the prior version of this theoretical model (de la Fuente, 2017). The previous model had only four levels of interaction and was more inaccurate (see Table 3). This means that the university teaching-learning process can be measured and classified along such a continuum.

In the case of the *second and third hypotheses*, the predictions were fulfilled quite accurately. The increase in the level of *self-regulation level* of students, significantly determined an increase in positive emotions (enjoyment, hope, pride…) and engagement, and a decrease in negative emotions (anger, anxiety, haplessness…), deactivation (boredom and relief) and burnout. On one hand, this lends empirical support to the construct of *self-regulation*, by showing that it has the potential to discriminate degrees of positive and negative emotions in students. This result is consistent with plentiful prior evidence that has shown a positive, significant correlation between self-regulation and the personality factor of *conscientiousness*, leading us to consider that self-regulation is a meta-behavioral variable that materializes this personality variable, associated with less stress, in contrast to the variable of *neuroticism* (Cheng et al., 2017). Also, consistent would be the expectancy-value theory (Pekrun, 2006; Stark et al., 2017), if we take degree of *self-regulation* as a correlate of a higher level of expectancy, of the task value, the effort and the success of university students (Garzón-Umerenkova et al., 2018).

On the other hand, there is evidence to support the construct of a *regulatory teaching level*, by establishing that this variable also determines the degree of students' positive and negative emotions, engagement and burnout. Thus, the positive emotion that is prompted by greater levels of external regulation is *confidence*—resulting from a more predictable context—while a negative emotion of *anger* or *hopelessness* results from the lack of contextual regulation or from dysregulation. These results are consistent with evidence-based recommendations and are required in order to implement the *regulatory teaching* or *effective teaching* (Roehrig et al., 2012): (1) Cognitive quality of the instruction task; (2) Quality of motivation during the instruction; (3) Support for autonomy through teaching self-regulation; (4) Goal structures, practices and performance expectations; (5) Design of tests and quizzes; (6) Performance consequences.

In general, this classification would reveal the interdependence between the self-regulation level, and regulatory teaching level, and type of academic emotionality. Greater levels of positive or negative emotionality, ultimately entail, greater experiences of engagement or burnout.

### Limitations and Future Directions

Beyond the evidence of the positive and negative emotionality that characterizes the interactions described above, there is still a need to establish whether the different interactions produce different specific stress factors coming from the context, therefore resulting in stress responses from the students. This aspect has not been addressed in the present research study. This would mean looking further into scientific evidence that would confirm the precise origin of positive or negative emotionality that stems from the teaching context. Future research studies should also establish the relationship between regulatory teaching and the teacher's own achievement emotions (Frenzel et al., 2016) or the emotional intelligence of the teacher (Pishghadam et al., 2017). It is plausible that a teacher who deploys a regulatory teaching process—taking into account his/her own high expectancy-value—will probably experience their teaching situation with positive emotionality, while a teacher who teaches in a non-regulating or dysregulating fashion will have greater negative emotionality. This interesting hypothesis should be tested in the future.

## Conclusions and Implications

### Conclusions

The most interesting result of this study has to do with the *cumulative or combined effect*, of the effects produced jointly by both variables. This is seen in the consistent, linear function that explains the combined effect of each variable on university students' emotional experience, as well as their place on the engagement-burnout continuum. This effect is especially important because it shows that all students benefit from external regulation, while they are also harmed by non-regulation or dysregulation. Similarly, it shows that students with high self-regulation call for and are more committed to highly regulatory contexts. This step forward in the interactive, contextualized study of students' achievement emotions represents progress toward consolidating contextualized (i.e., third level) *molar psycho-educational models* in real settings, and not only knowledge about relations between achievement emotions and personality variables, in *molecular-level models* (de la Fuente et al., 2019). This contribution allows us to more accurately and interactively reconceptualize the relative weight of variables pertaining to the subject and to the teaching context, when explaining university students' experiences with achievement emotions. A remarkable similarity was found between emotional experiences in the different situations -class, study and test- (Pekrun, 2006; Pekrun and Perry, 2014), which may suggest a certain *unified emotional experience* or an experience of the teaching-learning process as one overall stimulus.

### Implications for the Practice of Educational Psychology

Several implications from this investigation can be noted. First, it is important to know students' level of *self-regulation*, so that personalized intervention programs may be applied. If average levels of self-regulation in university students involve non-regulated behaviors (mid-level in positive and negative emotions) or dysregulated behaviors (high level of negative and low level of positive emotions), university guidance and counseling services ought to detect and help these types of students, as they begin their university studies, to promote stress management and coping strategies, and so minimize the impact of negative effects from the university experience. Certain programs in current use might help toward this end (de la Fuente, 2015c), either in a face-to-face format or through online technology tools (de la Fuente et al., 2018).

Second, there is an essential need to evaluate and know the level of effective teaching, and to detect the different contexts of regulatory teaching, especially when these are non-regulating or dysregulating, because of their negative emotional on students' emotional experiences. The academic experiences of emotional disconnection (boredom) and of negative emotionality (anger, anxiety, shame, hopelessness) have been amply associated with academic failure and dropout (Putwain et al., 2018, 2019; Reindla et al., 2018). There is also a need to observe experiences of satisfaction (enjoyment, hope, engagement), that these might be maintained and promoted within the university community. Therefore, university teacher training programs should include the knowledge, skills and attitudes of the teacher to promote positive emotions in students.

## Data Availability

The datasets generated for this study are available on request to the corresponding author.

## Ethics Statement

This studies involving human participants were reviewed and approved by Comité de Ética de la Investigación. Universidad de Navarra. The patients/participants provided their written informed consent to participate in this study.

## Author Contributions

JF has coordinated the R&D Project, has made the general design, data analysis, and first writing of the manuscript. JM-V has reviewed the design and analysis of data. FP-S has collected the data sample and has revised the manuscript. AG-U has collected the data sample and has revised the manuscript. MV has reviewed the previous evidence and the theoretical foundation. PP has provided the translation of the instruments and validated them in Spanish.

### Conflict of Interest Statement

The authors declare that the research was conducted in the absence of any commercial or financial relationships that could be construed as a potential conflict of interest.
