# Supplementary material for: Applying the SRL vs. ERL Theory to the Knowledge of Achievement Emotions in Undergraduate University Students
Source: Front Psychol. 2019 Sep 18;10:2070. doi: 10.3389/fpsyg.2019.02070 (PMC6760021; doi:10.3389/fpsyg.2019.02070)
Supplement: Supplementary file 1 [file Data_Sheet_1.pdf]

ANEX I. EXAMPLES OF ITEMS (COMPLEMENTARY MATERIAL)

Self-Regulation Questionnaire. SRQ (Brown, Miller & Lendonsky,1998; Short version: Pichardo, et al, 2014):

Goal setting
42. I set goals for myself and keep track of my progress.
47. Once I have a goal, I can usually plan how to reach it.
49. If I make a resolution to change something, I pay a lot of attention to how I am doing.
33. I have a hard time setting goals for myself.
1. I usually keep track of my progress toward my goals.
40. I have trouble making plans to help me reach my goals.

Perseverance
34. I have a lot of willpower.
6. I get easily distracted from my plans.
41. I am able to resist temptation.

Decision making
5. I have trouble making up my mind about things.
12. I put off making decisions.
19. When it comes to deciding about a change, I feel overwhelmed by the choice.
55 Little problems or distractions throw me off course.
13. I have so many plans that it is hard for me to focus on any one of them.

Learning from mistakes
21. I do not seem to learn from my mistakes.
28. I usually only have to make a mistake one time in order to learn from it.
57. I learn from my mistakes.

Interactive Assessment of the Teaching Learning Process, IATLP Scale (de la Fuente & Martínez-Vicente, 2007)

Regulatory Teaching (External regulation)

Part A. Teacher's General Behavior
1. At the beginning of each unit or lesson, the teacher explains why we are going to learn the material.
2. At the beginning of each activity, the teacher explains why we are going to do it.
3. The teacher explains the objectives of the activities we are going to carry out.
4. The teacher tries to determine whether the students have understood the learning objectives well.
5. The teacher presents the subject matter we are going to work on using some kind of conceptual map, diagram, chart, script, etc.
6. The teacher shows existing relationships between the material we are going to work on and other material we learned previously.
7. The teacher indicates which content items are the most important ones to learn in each unit or lesson.
8. The teacher makes the classes enjoyable.
9. The teacher is concerned that students feel comfortable in class.
10. The teacher is clear and orderly in his or her explanations.
11. The teacher frequently informs us as to our progress in the subject.
12. The teacher allows us to speak in class about how we are learning.

13. The teacher takes time to address our concerns or doubts.
14. The teacher helps us with corrections.
15. The teacher realizes when students have trouble learning a topic.
16. The teacher explains how we are going to be evaluated.
17. The teacher allows participation in evaluating his teaching.
18. The teachers makes us reflect on our learning in order to improve it.

Part C. Regulation Activities in Learning
30. The teacher does learning preparation activities with us.
31. The teacher presents a class work plan for each lesson or topic.
34. The teacher uses some activity (dialogue, questionnaire, etc.) to evaluate what we know while the lesson or topic is in progress.
35. The teacher uses some activity (dialogue, questionnaire, etc.) to evaluate what we know when we have finished the lesson or topic.
36. While we are learning, the teacher dialogues with the students about the objectives of the lesson or topic.
37. While we are learning, the teacher creates opportunities so we can think together about how we are learning.
38. While we are learning, the teacher helps us to make clear and realistic learning goals.
39. While we are learning, the teacher works with us on skills for reviewing and modifying our learning objectives.
40. While we are learning, the teacher makes us think about the way we are learning: at the beginning, during and after finishing the activities.
41. While we are learning, the teacher asks us to reflect, instead of making us repeat all the information.
42. While we are learning, the teacher makes us feel satisfied through learning “how to learn better”.

Achievement Emotions. The Achievement Emotions Questionnaire, AEQ (Goetz, Titz, & Perry, 2000)

Class
1. I get excited about going to class
9. I am full of hope
21. I feel scared
36. I get bored
43. I get embarrassed
70. I am proud of myself
73. I am angry

Learning
81. I look forward to studying
87. Because I´m bored I have no desire to learn
95. I feel hopeless when I think about studying
113. My sense of confidence motivates me
130. I feel helpless
153. I feel resigned

Tests
156. I look forward to the exam.
165. I feel sick to my stomach
188. I am very nervous
199. I feed humiliated
205. I feel hopeless

Engagement-Burnout Scale for Students (Shauffeli et al., 2002).

Engagement

Vigor
1. When I am studying, I feel mentally strong.
2. I can continue for a very long time when I am studying.
3. When I study, I feel like I am bursting with energy.
4. When studying I feel strong and vigorous.
5. When I get up in the morning, I feel like going to class.

Dedication
1. I find my studies to be full of meaning and purpose.
2. My studies inspire me.
3. I am enthusiastic about my studies.
4. I am proud of my studies.
5. I find my studies challenging.

Absorption
1. Time flies when I am studying.
2. When I am studying, I forget everything else around me.
3. I feel happy when I am studying intensively.
4. I can get carried away by my studies

Burnout:

Exhaustion
1. I feel emotionally drained by my studies.
2. I feel used up at the end of a day at university.
3. I feel tired when I get up in the morning and I have to face another day at the university.
4. Studying or attending a class is really a strain for me.
5. I feel burned out from my studies.

Cynicism
1. I have become less interested in my studies since my enrollment at the university.
2. I have become less enthusiastic about my studies.
3. I have become more cynical about the potential usefulness of my studies.
4. I doubt the significance of my studies.

Efficacy
1. I can effectively solve the problems that arise in my studies.
2. I believe that I make an effective contribution to the classes that I attend.
3. In my opinion, I am a good student.
4. I feel stimulated when I achieve my study goals.
5. I have learned many interesting things during the course of my studies.
6. During class I feel confident that I am effective in getting things done
